# Unusual Presentation of Bladder Cancer in a Young Male With Significant Tattoo Exposure: A Case Report

**DOI:** 10.7759/cureus.69637

**Published:** 2024-09-18

**Authors:** Manuel Pizarro-Mondesir, Claudia Ramirez-Marcano, Ricardo Arriaga-Perry, Vincent Rodriguez-Bury

**Affiliations:** 1 Urology, Universidad Central del Caribe School of Medicine, Bayamón, PRI; 2 Psychiatry, Universidad Central del Caribe School of Medicine, Bayamón, PRI; 3 Urology, Doctors’ Center Hospital, Carolina, PRI

**Keywords:** bladder cancer, cystoscopy, high grade urothelial carcinoma, polycyclic aromatic hydrocarbons, tattoo ink, trans urethral resection of bladder tumor (turbt), transurethral resection of bladder tumor (turbt), urothelial bladder cancer, young adult male

## Abstract

Recent studies have shown that polycyclic aromatic hydrocarbons (PAHs) can be found in commercial black tattoo inks raising suspicion of tattoo-related PAHs exposure to cancer risk. We present a case of a 27-year-old Hispanic nonsmoker male with bladder cancer (BC) following extensive tattoo sessions totaling over 100 hours. The patient was treated with transurethral resection of the bladder tumor (TURBT) and adjuvant intravesical Bacillus Calmette-Guerin (BCG) therapy. Although the oncogenesis of urothelial tumors in young patients is unclear, multiple environmental and genetic factors may contribute to the etiology. This case report underscores the importance of conducting toxicological and epidemiological studies on PAHs and emphasizes the need for increased documentation of tattoos in patients diagnosed with BC.

## Introduction

Bladder cancer (BC) is the 10th most common cancer worldwide and is the sixth most incident neoplasm in the United States [1]. The risk of developing BC increases with age. For men over 70, the probability is 3.7%, compared to 0.92% for men aged 60 to 69, and 0.38% for those aged 40 to 59 [2]. Considerable investigations have shown that the primary modifiable risk factors for BC are cigarette smoking and occupational exposure to carcinogens [3].

Polycyclic aromatic hydrocarbons (PAHs) are hazardous chemical pollutants produced by the incomplete combustion of organic materials such as tobacco, wood, coal, petroleum products, and even during food preparation [4]. Humans can be exposed to PAHs through various routes, including inhalation, dermal contact, and ingestion [4]. Many PAHs are recognized as carcinogens, mutagens, and teratogens. Studies have proven that long-term exposure to PAH increases the risk of BC due to its highly toxic, carcinogenic mechanisms [4]. Recent studies have shown that PAH can also be found in commercially available black tattoo inks raising suspicion of tattoo-related PAH exposure to cancer risk, particularly in younger populations [5]. This case report presents a case of urothelial carcinoma on a 27-year-old male with over 100 hours of tattoo sessions as the only known risk factor.

## Case presentation

The patient is a 27-year-old Hispanic male who presented to the outpatient urologic clinic with the complaint of novel onset gross painless hematuria persistent for the past month. The patient, otherwise healthy and a non-smoker, reported heavy alcohol consumption and currently works in construction with a gypsum board. During the interview, we noticed a history of tattoos that began nine years ago. A family history of Lynch syndrome, hereditary nonpolyposis colorectal cancer, and other malignancies was denied. The patient denied experiencing dysuria or voiding problems. Physical examination revealed no remarkable findings aside from tattoos covering both arms, left leg, chest, and abdomen. Table 1 illustrates the patient's laboratory findings, showcasing large amounts of blood and an elevated red blood cell count of >100/hpf on urinalysis.

**Table 1:** Patient's urinalysis H: high; WBC: white blood cell; RBC: red blood cell; hpf: high-power field; lpf: low-power field; E.U./dL: Ehrlich units per deciliter

Test	Observed value	Reference range
Color	Yellow	Yellow-amber
Appearance/Clarity	Clear	Clear
Blood	Large (H)	Negative
Glucose	Negative	Negative
Bilirubin urine	Negative	Negative
Ketone	Negative	Negative
Specific gravity	1.022	1.005-1.030
pH	7.0	4.6-8.0
Protein	Negative	Negative
Urobilinogen	1.0	0.2-1.0 E.U./dL
Nitrite	Negative	Negative
Leukocyte esterase	Negative	Negative
WBC	3-5	0-5/hpf
RBC	>100	0-5/hpg
Squamous epithelial cells	Few	None-Few
Hyaline casts	0-2	0-8/lpf
Bacteria	Few	None-Few

**Figure 1:**
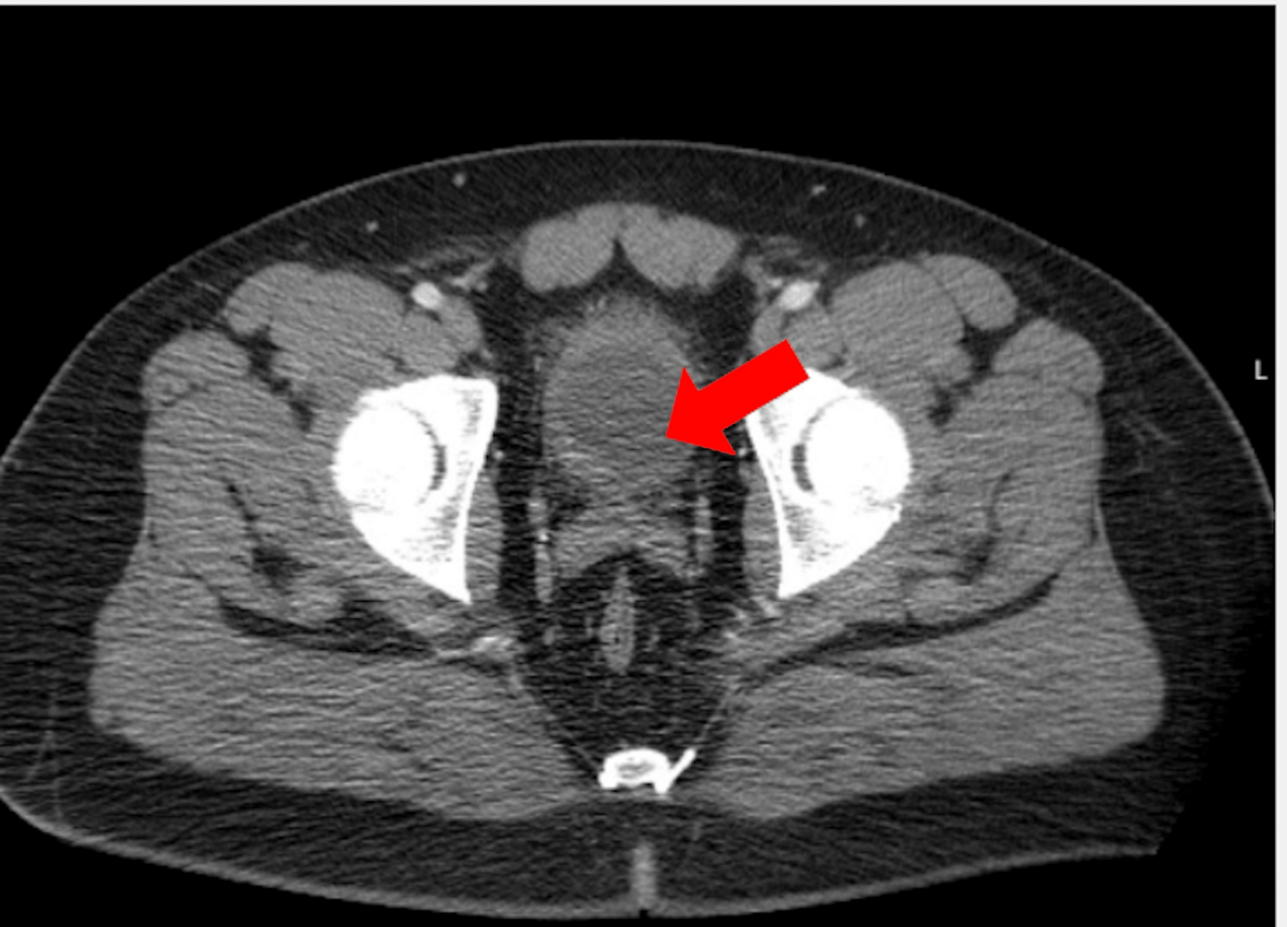
Contrast axial abdominopelvic CT scan demonstrating a bladder mass suggestive of malignancy. The red arrow in the figure is pointing at diffuse wall thickening involving the anterior aspect of the bladder with subtle pre-vesical fat stranding suggestive of malignancy.

The urine culture returned negative after 48 hours of incubation. Subsequently, a renal sonogram revealed both kidneys at their usual position with normal bilateral renal cortical echogenicity with no evidence of hydronephrosis or kidney stones on either side. Contrast abdominopelvic CT scan reported diffuse wall thickening involving the anterior aspect of the bladder with subtle pre-vesical fat stranding suggestive of malignancy (Figure 1).

A cystoscopy revealed a large papillary bladder mass at the right bladder neck. Subsequent transurethral resection of the bladder tumor (TURBT) was performed. During surgery, the patient was placed in a lithotomy position and the genitalia was prepped and draped. Then, a 26Fr cystoscope was introduced with a direct vision instrument. After landmark identification, and with the aid of an Iglesias instrument and a bipolar cutting loop, a 5cm papillary bladder mass located at the right bladder neck was resected. Fragments were then removed with a Toomey syringe and sent to pathology. Second-look cystoscopy and fulguration were performed. A 20Fr two-way Foley catheter was left in place. The patient was transferred to the recovery room in stable condition.

**Figure 2:**
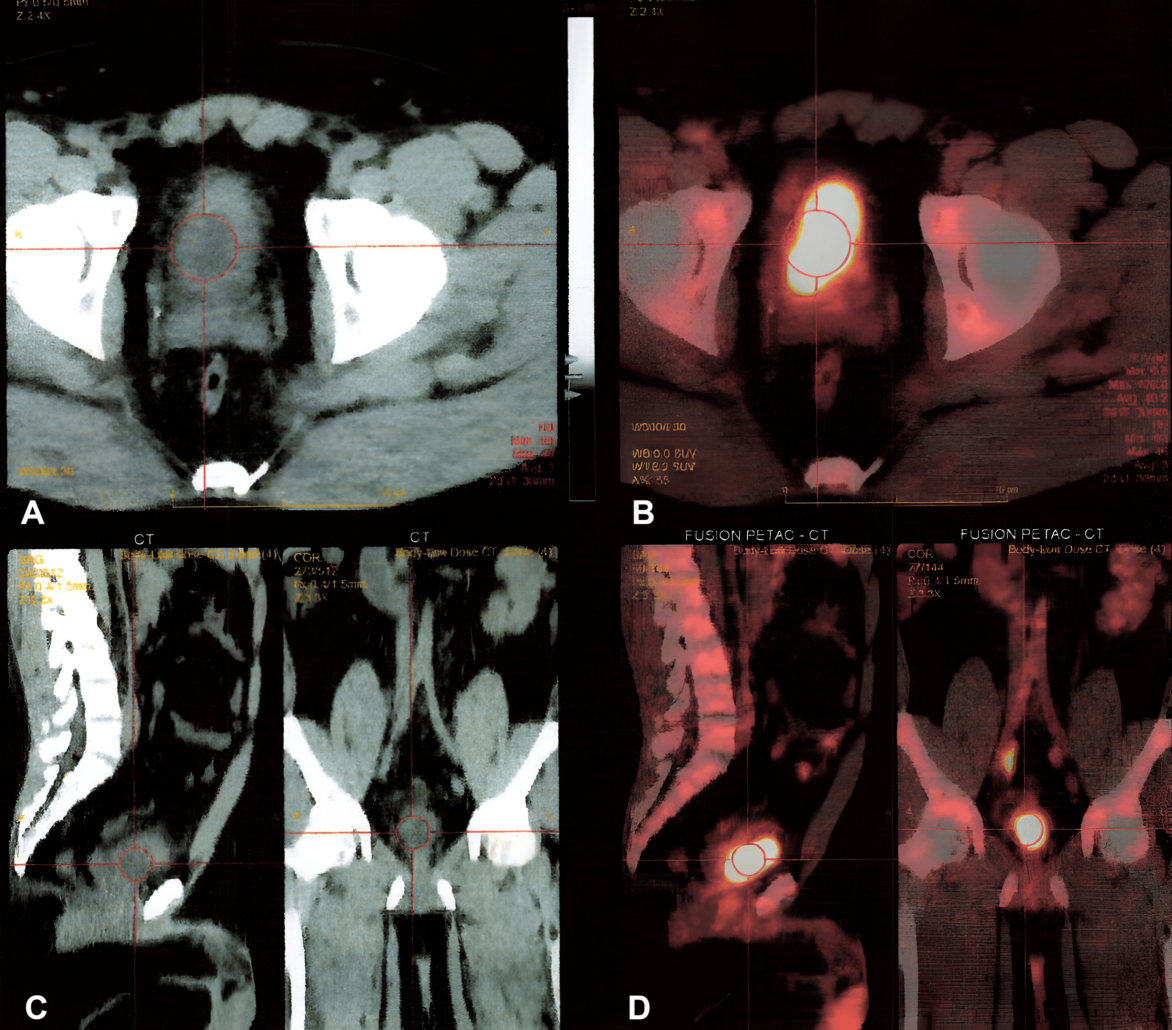
Fused PET/CT images with no evidence of metastatic disease per PET criteria. After intravenous administration of F-18 fluorodeoxyglucose (FDG), a non-contrast multi-detector CT scan was performed at 5mm intervals contiguous axial images for anatomical correlation and attenuation correction purposes only. A series of overlapping positron emission tomography (PET) images were then obtained 60 minutes after FDG injection. Label A - CT transverse (axial) slice - red circle pointing to concentric thickening of the bladder wall with no definite focal FDG avid lesion. Label B - fusion PET/CT transverse (axial) slice - red circle pointing to concentric thickening of the bladder wall with no definite focal FDG avid lesion. Label C - CT sagittal and coronal slice - red circle pointing to concentric thickening of the bladder wall with no definite focal FDG avid lesion. Label D - Fusion PET/CT sagittal and coronal slice - red circle pointing to concentric thickening of the bladder wall with no definite focal FDG avid lesion.

Histopathological examination showed a high-grade, invasive papillary urothelial carcinoma, pT1. Consequently, the patient underwent a second TURBT. The pathology revealed urothelial dysplasia at the dome and right bladder wall with muscle present and not involved with the tumor. Positron emission tomography (PET) scan was negative for metastases (Figure 2).

Post-resection, the patient remains well with a complete response after a six-week course of intravesical Bacillus Calmette-Guerin (BCG) therapy. Cystoscopy revealed inflammation at the bladder dome with calcifications. Following the American Urological Association (AUA) guidelines, a high-risk patient who fully responds to induction BCG should continue maintenance BCG for three years, as tolerated. The typical maintenance schedule involves weekly triplets of BCG administered at 3, 6, 12, 18, 24, 30, and 36 months from the start of induction therapy. The regimen, based on the Lamm/SWOG protocol, has shown a 19% improvement in five-year recurrence-free survival (from 41% to 60%) and a 6% improvement in five-year worsening-free survival for patients who adhered to the schedule [6]. Given the patient’s high-risk features and age, a third cystoscopy and biopsy/TURBT have been scheduled in the operating room to ensure there is no residual cancer.

## Discussion

BC is largely influenced by external risk factors, where approximately 4.3% of patients have a first-degree relative with BC, and up to 50% of patients have a family history of cancer [7]. Tobacco smoking contributes to around 50% of BC cases [7]. The role of occupational agents in the development of BC has been documented since the late 19th century and has been extensively studied [8]. In 1989, Bonassi et al. documented that workplace exposure to PAHs and aromatic amines (AAs) are significant risk factors for BC [8]. Cassidy et al. later observed a notable increase in BC risk with longer employment durations. Specifically, jobs with less than 10 years of exposure showed no significant risk increase, whereas extending employment beyond 10 years significantly raised the risk [3]. Elevated risks were also noted in various occupations, including medicine and health, waitstaff and bartenders, electrical assembly and installation, food and beverage preparation and service, motor freight, and structural work, among others [3].

PAHs enter the human body through the respiratory tract, skin, and digestive tract [9]. Once inside, they are transported to the lymph nodes, circulate in the bloodstream, and are metabolized primarily in the liver and kidneys [9]. PAHs can affect the bladder similarly to how they affect the lungs. This occurs through metabolic activation, which involves the induction of mono-oxygenase enzymes and the formation of covalent bonds with DNA, marking the initial stage of carcinogenesis [8]. PAHs reach the bladder as both parent compounds and their metabolites [8]. The bladder epithelium, with its high metabolic activity, can bind PAHs to its cellular DNA [8]. Predominantly, PAHs are excreted in bile and urine, with their hydroxylated metabolites (OHPAHs) conjugated to water-soluble glucuronic acid and sulfate for elimination [9].

Tattoo artists introduce tattoo ink inside the skin surface, inadvertently exposing individuals to a wide range of unknown ingredients, including AAs, heavy metals, and PAHs [10]. AAs can form within the skin due to reductive cleavage of organic azo dyes [10]. Additionally, heavy metals, namely, cadmium, lead, mercury, antimony, beryllium, and arsenic are associated with various health issues, including cancer, neurodegenerative diseases, cardiovascular, gastrointestinal, lung, kidney, liver, endocrine, and bone diseases [10]. PAHs, such as benzo(a)pyrene, are present in carbon black ink [5,10]. Lehner et al. documented levels of up to 200μg/g of PAH and up to 385μg/g of phenol in commercially available tattoo inks [5]. For a single black tattoo with a typical size of 400cm^2^, they estimated an amount of up to 400μg PAH and 770μg phenol are injected into the skin, thus highlighting a new pathway PAH intake in humans [5].

After tattooing and during wound healing, tattoo ink ingredients may persist in the skin or distribute throughout the human body, especially to regional lymph nodes [5]. PAHs in tattoo inks can either bind to carbon black or dissolve in the ink's suspension solvents [5]. In lymph nodes, these PAH molecules could potentially be released from carbon black to varying degrees and over an uncertain period [5]. Moreover, black ink particles may also accumulate in other organs such as the liver, spleen, and kidneys, depending on their transportation route via the lymphatic or blood vessel systems. This is of great importance because PAHs may have deleterious effects elsewhere in the human body.

In this case report, we highlight a de novo urothelial carcinoma in a young Hispanic adult male who is a nonsmoker with no remarkable family history of cancer. Even though his occupation as a construction worker carries a potential risk for the development of BC, he has only been working in this field for two years. Although the oncogenesis of urothelial tumors in young patients is unclear, multiple environmental and genetic factors may contribute to the etiology. Interestingly, our patient underwent more than 100 hours of tattoo sessions, resulting in large tattoos covering both arms, left leg, chest, and abdomen. Although there is no data directly linking tattoo ink exposure to BC, extensive research has established a clear correlation between PAH exposure and BC. Therefore, we can hypothesize that the high levels of PAHs found in tattoo ink, combined with the number of tattoos this patient has, could be a contributing risk factor for his BC.

## Conclusions

The patient, a 27-year-old Hispanic man and nonsmoker, exhibited no discernible occupational risk factors, highlighting the possibility of both idiopathic and yet-to-be-identified risk factors contributing to the onset of BC. The occurrence of this cancer in this young patient with no known risk factors prompts the question, "Can PAH exposure via tattoo ink injection be considered a risk factor for urothelial carcinoma?" Besides inhalation and ingestion, tattooing has proven to be an additional, direct, and effective route of PAH uptake into the body. This case report underscores the importance of conducting toxicological and epidemiological studies on PAHs and emphasizes the need for increased documentation of tattoos in patients diagnosed with urothelial carcinoma. Currently, the lack of direct evidence linking tattoos to BC highlights the need for more research in this area. Our case report on a young patient with no significant risk factors for BC, yet a history of extensive tattooing, could offer valuable insights into this emerging field. It proposes that exposure to PAH in tattoo inks might be a potential new risk factor, highlighting the need for further investigation into the systemic effects of these substances.
